# Tear cytokine dataset in patients with allogeneic stem cell transplant treated with topical cyclosporine

**DOI:** 10.1016/j.dib.2026.112704

**Published:** 2026-03-19

**Authors:** Jingwen Yu, Li Lim, Yu-Chi Liu, Yeh Ching Linn, Louis Tong

**Affiliations:** a Ocular Surface Research Group, Singapore Eye Research Institute, Singapore; b Corneal and External Eye Disease Service, Singapore National Eye Centre, Singapore; c Eye-Academic Clinical Program, Duke-National University of Singapore Medical School, Singapore; d Department of Haematology, Singapore General Hospital, Singapore; e Department of Ophthalmology, Yong Loo Lin School of Medicine, National University of Singapore, Singapore

**Keywords:** Allogeneic hematopoietic stem cell transplant, Cyclosporine-a, Tear disorder, Cytokine

## Abstract

This article showed the data of ocular clinical parameters, tear fluid cytokines, and topical cyclosporine-A 0.1% (Ikervis®) compliance in allogeneic hematopoietic stem cell transplant (allo-HSCT) patients. Study visits were conducted at 3–5 weeks pre-allo-HSCT (screening), pre-allo-HSCT, and 3/6/12 months post-allo-HSCT, with data collected at each time point. At each visit, tear samples were assessed for 96 cytokines by the Proximity Extension Assay O-linked target 96 platform.

Specifications Table

**Table:** 

Subject	Health Sciences, Medical Sciences & Pharmacology
Specific subject area	Tear cytokine in allo-HSCT patients
Type of data	Excel (.xlsx format)
Data collection	Data were collected from Singapore General Hospital allo-HSCT patients (Nov 2019–Sep 2021 at five visits. Tear volume and samples were collected via the Schirmer I test (Clement Clark®, Essex, UK). Ocular surface assessments were performed as previously described [1] Briefly, the assessment included corneal staining, non-invasive tear break-up time (NIBUT), conjunctival redness and tear osmolarity. Dry eye symptoms were evaluated via the Standardized Evaluation of Eye Dryness (SPEED) questionnaire. Compliance with the use of Ikervis® was based on the daily drug diary [2].
Data source location	Country: Singapore Institution: Singapore National Eye Centre
Data accessibility	Repository name: Mendeley Data Data identification number: doi: 10.17632/643wd79h3j.4 Direct URL to data: https://data.mendeley.com/datasets/643wd79h3j/4
Related research article	Tong L, Liu YC, Yeo SWJ, Liu C, Lee IXY, Linn YC, Ho A, Than H, Quek JKS, Hwang WYK, Lim FLWI, Lim L. Tear Cytokine Changes up to One Year After Allogeneic Hematopoietic Stem Cell Transplant: Effect of Daily Topical Cyclosporine-A 0.1% Emulsion. Int J Mol Sci. 2025 Jun 19;26(12):5915. doi: 10.3390/ijms26125915.

## 1 Value of the Data

• Here we report the ocular clinical parameters and tear cytokine data of allo-HSCT patients using topical cyclosporine-A 0.1% (Ikervis®) from 3 to 5 weeks prior to allo-HSCT to 12 months after allo-HSCT.
• These precious data are not easy to acquire (only publications involved are cross-sectional studies) and will help researchers to understand the temporal changes of tear cytokines in allo-HSCT patients. Once the data are publicly available, some investigators may use the data for further analysis.
• It is still not fully understood that why the immune system selectively targets ocular tissues after HSCT, even years later, and even sparing all other organs in the late stage. This data (on 93 cytokines), which is collected up to one year after HSCT, may shed light on the medical mystery, because cytokines provide information about the different arms of the immune system and its activity.

## 2 Background

Dry eye disease (DED) is a common ocular complication of graft-versus-host disease (GVHD) after allo-HSCT. Topical cyclosporine (CsA) is safe and effective for treating GVHD-linked DED, but data on its use for ocular GVHD prophylaxis is limited. Since tear cytokines drive ocular inflammation, this dataset aimed to show 1-year tear cytokine changes in allo-HSCT patients with topical cyclosporine-A.

## 3 Data Description

The dataset comprises longitudinal ocular clinical parameters and tear cytokine measurements collected from patients undergoing allogeneic hematopoietic stem cell transplantation (allo-HSCT). A total of 13 patients were included in this cohort. Data were collected at up to five study timepoints for each patient: 3–5 weeks pre-allo-HSCT (screening), immediately pre-allo-HSCT, and 3, 6, and 12 months post-allo-HSCT. At each visit, data were recorded separately for each eye, resulting in a repeated-measures, longitudinal dataset.

Tear cytokine analysis was performed using the Olink® Target 96 Proximity Extension Assay (PEA) platform, yielding measurements for up to 96 analytes per sample. In addition to cytokine data, the dataset includes demographic information, disease characteristics, treatment-related variables, and ocular surface clinical parameters such as non-invasive tear break-up time, corneal staining, conjunctival redness, tear osmolarity, and dry eye symptom scores.

The metadata file (metadata.xlsx) serves as a data dictionary and provides detailed descriptions of all variables included in the dataset. This file documents variable names, definitions, data types, units of measurement, associated data files and worksheets, and corresponding study timepoints. No individual-level measurements are contained in this file.

The processed dataset file (processed_data.xlsx) contains the final data used in the associated publication. Each row represents a single eye at a specific study visit. This file includes de-identified patient identifiers, visit timepoints, eye laterality, ocular clinical parameters, and tear cytokine values. Cytokine measurements in this file have been corrected for equivalent tear volume based on Schirmer strip wetted length, allowing direct statistical analysis of longitudinal changes.

The raw Olink output file (olink_raw_output.xlsx) contains the original NPX values generated directly by the Olink Target 96 platform prior to tear volume correction or downstream processing. This file also includes assay-related information and quality control outputs produced by the Olink software. These raw data are provided to enable independent reprocessing, alternative normalization strategies, and reproducibility of cytokine analyses.

Together, these files provide a comprehensive and transparently organized resource that supports longitudinal analysis of tear cytokine dynamics and ocular surface changes following allo-HSCT.

## 4 Experimental Design, Materials and Methods

Thirteen patients received topical cyclosporine-A 0.1% (Ikervis®, Santen Pharmaceuticals) once daily to both eyes and preservative-free sodium hyaluronate 0.3% (Hialid Mini, Santen Pharmaceuticals) pro re nata (PRN) from 3to 5 weeks before allo-HSCT to 12 months after allo-HSCT, plus systemic GVHD therapy. Study visits occurred at 3–5 weeks pre-allo-HSCT (screening), immediately pre-allo-HSCT, and 3 months, 6 months, and 12 months post-allo-HSCT, with ocular clinical evaluations performed as reported earlier [1].

Tear samples were collected using 5-minute Schirmer strips and frozen at −80 °C. For tear extraction, Schirmer strips with tear fluid were submerged in the elution buffer and then centrifuged to collect clear supernatants. For cytokine analysis, 96-panel cytokines were assayed via the Proximity Extension Assay O-linked target 96 platform (Olink® Target 96, Thermo Fisher Scientific, USA) as previously reported [3]. Briefly, each oligonucleotide antibody pair contains unique DNA sequences, allowing hybridization only to each other. Subsequent proximity extension created 96 unique DNA reporter sequences (amplified by real-time PCR).

The protein concentration was accounted for by correcting the raw data against each eye’s equivalent tear volume. Tear volume was determined through tear elution from their Schirmer strips. The tear equivalent volume was standardized by relating the fixed wetted length of the strips to a fixed volume. This allowed the creation of a linear expression for the interpolation for different wetted lengths.

**Table:** Calibration of Schirmer strip wetted length to tear volume.

Fixed Volume (uL)	Schrimer Strip Length (mm)
2	1
4	5
6	7
8	10
15	17
20	24
25	30
30	35



Furthermore, the following equation was used to obtain the corrected concentration of each analyte:

$$\begin{array}{ccc} Corrrected\;concentration\;of\;analyte & = & \;\frac{Equivalent\;Tear\;volume\;}{300+Equivalent\;Tear\;Volume\;}\\ & & \times Raw\;concentration\;of\;analyte \end{array}$$

We have adopted the same calculation and elution methods for our previous published paper [4].

## Limitations

The sample size is small, and the follow-up period only lasted until one year after transplantation.

## Ethics Statement

Approval for the study was granted by the Institutional Review Board of SingHealth (reference number 2019/2635), Singapore, and the study was conducted in accordance with the Declaration of Helsinki and written informed consent was obtained from the subjects.

All data shared in this repository are fully de-identified. The publicly shared dataset does not contain any information that could be used to identify individual participants.

## Data Availability

Mendeley DataCytokines in Patients with Allogeneic Stem Cell Transplant Treated with Topical Cyclosporine (Original data).
